# Side-channel attacks against the human brain: the PIN code case study (extended version)

**DOI:** 10.1186/s40708-018-0090-1

**Published:** 2018-10-29

**Authors:** Joseph Lange, Clément Massart, André Mouraux, François-Xavier Standaert

**Affiliations:** UCLouvain, 1348 Louvain-la-Neuve, Belgium

**Keywords:** Brain–computer interfaces (BCIs), Electroencephalography (EEGs), Security, Privacy

## Abstract

We revisit the side-channel attacks with brain–computer interfaces (BCIs) first put forward by Martinovic et al. at the USENIX 2012 Security Symposium. For this purpose, we propose a comprehensive investigation of concrete adversaries trying to extract a PIN code from electroencephalogram signals. Overall, our results confirm the possibility of partial PIN recovery with high probability of success in a more quantified manner and at the same time put forward the challenges of full/systematic PIN recovery. They also highlight that the attack complexities can significantly vary in function of the adversarial capabilities (e.g., supervised/profiled vs. unsupervised/non-profiled), hence leading to an interesting trade-off between their efficiency and practical relevance. We then show that similar attack techniques can be used to threat the privacy of BCI users. We finally use our experiments to discuss the impact of such attacks for the security and privacy of BCI applications at large, and the important emerging societal challenges they raise.

## Introduction

*State of the art* The increasing deployment of Brain–computer interfaces (BCIs) allowing to control devices based on cerebral activity has been a permanent trend over the last decade. While originally specialized to the medical domain (e.g., [1, 2]), such interfaces can now be found in a variety of applications. Notorious examples include drowsiness estimation for safety driving [3] and gaming [4]. Quite naturally, these new capabilities come with new security and privacy issues, since the signals BCIs exploit can generally be used to extract various types of sensitive information [5, 6]. For example, at the USENIX 2012 Security Symposium, Martinovic et al. showed empirical evidence that electroencephalogram (EEG) signals can be exploited in simple, yet effective attacks to (partially) extract private information such as credit card numbers, PIN codes, dates of birth and locations of residence from users [7]. These impressive results leveraged a broad literature in neuroscience, which established the possibility to extract such private information (e.g., see [8] for lie detection and [9] for neural markers of religious convictions). Or less invasively, they can be connected to linguistic research on the reactions of the brain to semantic associations and incongruities (e.g., [10–12]). All these threats are gaining relevance with the availability of EEG-based gaming devices to a general public [13, 14].

*Motivation and goals* Based on this state of the art, the next step is to push the evaluation of the side-channel threat model in the context of BCI-based applications further. In this respect, the seminal work of Martinovic et al. clearly puts forward the existence of an exploitable bias for various types of private information extraction. But quantifying the impact of this bias in concrete adversarial contexts was left as an important challenge. Typical questions include:

- Can we exactly extract private information with high success rate by increasing the number of observations in side-channel attacks exploiting BCIs?
- How does the effectiveness of unsupervised (aka non-profiled) side-channel attacks exploiting BCIs compare to supervised (aka profiled) ones?
- How efficiently can an adversary build a sufficiently accurate model for supervised (aka profiled) side-channel attacks exploiting BCIs?
- How similar/different are the behavior and the resistance of different users in the context of side-channel attacks exploiting BCIs?

Interestingly, these are typically questions that have been intensively studied in the context of side-channel attacks against cryptographic devices (see [15] for an engineering survey and the proceedings of the CHES conference for regular advances in the field [16]). In particular, a recurring problem in the analysis of such implementations is to determine their worst-case security level, in order to bound the probability of success of any adversary in the most accurate manner [17]. This implies very different challenges than in the standard cryptographic setting, since the efficiency of such physical attacks highly depends on the adversary’s understanding and knowledge of his target device. Hence, a variety of tools have been developed in order to ensure that side-channel security evaluations are “good enough” (as described next). Our goal in this paper is to investigate the applicability of such tools in order to answer the previous questions regarding the efficiency and impact of side-channel attacks against the human brain.

*Contributions* For this purpose, we propose an in-depth study of (a variation of) one of the case studies in [7], namely side-channel PIN code recovery attacks, that share some similarities with key recovery attacks against embedded devices. In this respect, our contributions are threefold. After a description of our experimental settings (Sect. 2), we first describe a methodology allowing us to analyze the informativeness of EEG signals and their impact on security with confidence (Sect. 3). While this methodology indeed borrows tools from the field of side-channel attacks against cryptographic implementations, it also deals with new constraints (e.g., the limited amount of observations available for the evaluations and the less regular distribution of these observations, for which a very systematic and principled approach is particularly important). Second, we provide a comprehensive experimental evaluation of our side-channel attacks against the human brain using this methodology (Sect. 4). We combine information-theoretic and security analyses in the supervised/profiled and unsupervised/non-profiled contexts, provide quantified estimates for the complexity of the attacks and pay a particular attention to the stability of and confidence in our results. Eventually, and after a brief excursion toward the privacy issues raised by our experiments (i.e., what happens if the adversary aims to recover the user IDs rather than the PIN codes?), we conclude by discussing consequences for the security and privacy of BCI-based applications and list interesting scopes for further research (Sect. 6).

Admittedly, and as will be detailed next, our results can be seen as positive or negative. That is, we show in the same time that partial information about PINs can be extracted with confidence and that full PIN extractions are challenging because of the high cardinality of the target and risks of false positive. So they should mostly be viewed as a warning flag that such partial information is possible and may become critical when the cardinality of the target decreases and/or large amounts of data are available to the adversary.^1^

## Experimental setting and threat model

In our experiments, eight people (next denoted as users) agreed to provide the 4-digit PIN code that they consider the most significant to them, meaning the one they use the most frequently in their daily life. This PIN code was given by the users before the experiment started, stored during the experiment and deleted afterward for confidentiality reasons. Five other random 4-digit codes were generated for each user (meaning a total of six 4-digit codes per user).

Each (real or random) PIN was then shown on a computer exactly 150 times to each user (in a random order), meaning a total of 900 events for which we recorded the EEG signal in sets of 300, together with a tag *T* ranging from 1 to 6 (with $T=1$ the correct PIN and $T=2$ to 6 the incorrect ones). We used 32 Ag–AgCl electrodes for the EEG signals collection. These were placed on the scalp using a WaveGuard cap from Cephalon, using the international 10-10 system. The stimulus onset asynchrony (SOA) was set to 1.009 s (i.e., slightly more than 1 s, to reduce the environmental noise). The time each PIN was shown was set to 0.5 s. When no PIN was displayed on the screen, a + sign was maintained in order to keep the focus of the user on the center of the screen. We additionally ensured that two identical 4-digit codes were always separated by at least two other 4-digit codes. The split of our experiments in sub-experiments of 300 events was motivated by a maximum duration of 5 min, during which we assumed the users to remain focused on the screen. The signals were amplified and sampled at a 1000 Hz rate with a 32-channel ASA-LAB EEG system from Advanced NeuroTechnologies. Eventually, and in order to identify eye blinks which potentially perturb the EEG signal, we added two bipolar surface electrodes on the upper left and lower right sides of the right eye and rejected the records for which such an artifact was observed. This slightly reduced the total number of events stored for each user. (Precisely, this number was reduced to 900, 818, 853, 870, 892, 887, 878, 884, for users 1–8.)

This simplified setting naturally comes with limitations. First and concretely, the number of possible PIN codes for a typical smart card would of course be much larger than the 6 ones we investigate (e.g., 10,000 for a 4-digit PIN). In this respect, we first insist that the primary goal of the following experiments is to investigate the information leakages in EEG signals thoroughly, and this limited number of PIN codes allowed us to draw conclusions with good statistical confidence. Yet, we also note that this setting could be extended to a reasonable threat model. For example, one could target $\approx 1000$ different users by repeatedly showing them $\approx 10$ PIN codes among the 10,000 possible ones and recover one PIN with good confidence. Second, and since the attacks we carry out essentially test familiar versus unfamiliar information, there is also a risk of false positives (e.g., an all zero code or a close to correct code). In this respect, our mitigation plan is to exploit statistical tools minimizing the number of false negatives, therefore potentially allowing enumeration among the most likely candidates [18].

## Methodology

In this section, we describe the methodology we used in order to assess and better quantify the feasibility of side-channel attacks against the human brain. Concretely, and contrary to the case of embedded devices where the leakage distributions are supposed to be stable and the number of observations made by the adversary can be large, we deal with a very different challenge. Namely, we need to cope with irregular distributions possibly affected by outliers and can only assume a limited number of observations.

As a result, the following sections mainly aim to convince the reader that our treatment of the EEG signals is not biased by dataset-specific overfitting. For this purpose, our strategy is twofold. First, we apply the same (pre)processing methods to the measurements of all the users. This means the same selection of electrodes, the same dimensionality reduction and probability density function (PDF) estimation tools (with identical parameters), and the same outliers definition. Second, we systematically verified that our results were in the same time consistent with neurophysiological expectations and stable across a sufficient range of (pre)-processing parameters. As a result, our primary focus is on the confidence in and stability of the results, more than on their optimality (which is an interesting scope for further research). In other words, we want to guarantee that EEG signals provide exploitable side-channel information for PIN code recovery and to evaluate a sufficient number of observations for which such an attack can be performed with good success probability.

### Notations

We denote the (multivariate) EEG signals of our experiments with a random variable $\varvec{O}$, a sample EEG signal as $\varvec{o}$, and the set of all the observations available for evaluation as $\mathcal {O}$. These observations depend on (at least) three parameters: the user under investigation, next denoted with a random variable *U* such that $u\in \{1,2,\ldots ,8\}$; the nature of the 4-digit code observed (i.e., whether it is correct or a random PIN), next denoted with a random variable *P* such that $p\in \{0,1\}$; and a noise random variable *N*. Each observation is initially made of 32 vectors of 1000 samples, corresponding to 32 electrodes and $\approx 1$s per event.

### Supervised (aka profiled) evaluation

In order to best evaluate the actual informativeness of the EEG signals regarding the PIN displayed in our experiments and inspired by the worst-case side-channel security evaluations of cryptographic devices, our work first investigates so-called profiled attacks, which correspond to a supervised machine learning context. For this purpose, a part of the observations in $\mathcal {O}$ are used to estimate a (probabilistic) model $\hat{\Pr }_{\mathrm {model}}[P=p|\varvec{O}=\varvec{o}]$. The adversary/evaluator then uses this model in order to try extracting the PIN from the remaining observations. Note that our profiling is based on the binary random variable *p*, where $p=0$ if the PIN is random and $p=1$ if the PIN is real, and not based on the value of the PIN tag itself. This is motivated by the following practical and neurophysiological reasons:

- From a practical point of view, building a model for all the PINs and users seems impractical in real-world settings: this would require being able to collect multiple observations for each of the 10,000 possible values of a 4-digit code. Furthermore, and as discussed in Sect. 3.3, our real versus random profiling allowed us to lean toward realistic (non-profiled) attacks.
- From a neurophysiological point of view, the information we aim to extract is based on event-related potentials (ERPs) that have been shown to reflect semantic associations and incongruities [10–12]. In this respect, while we can expect a user to react differently to real and random 4-digit codes, there is no reason for him to treat the random codes differently. (Up to problems due to the apparition of other “significant” values that may lead to false positives, as will be discussed next.)

**Fig. 1:**
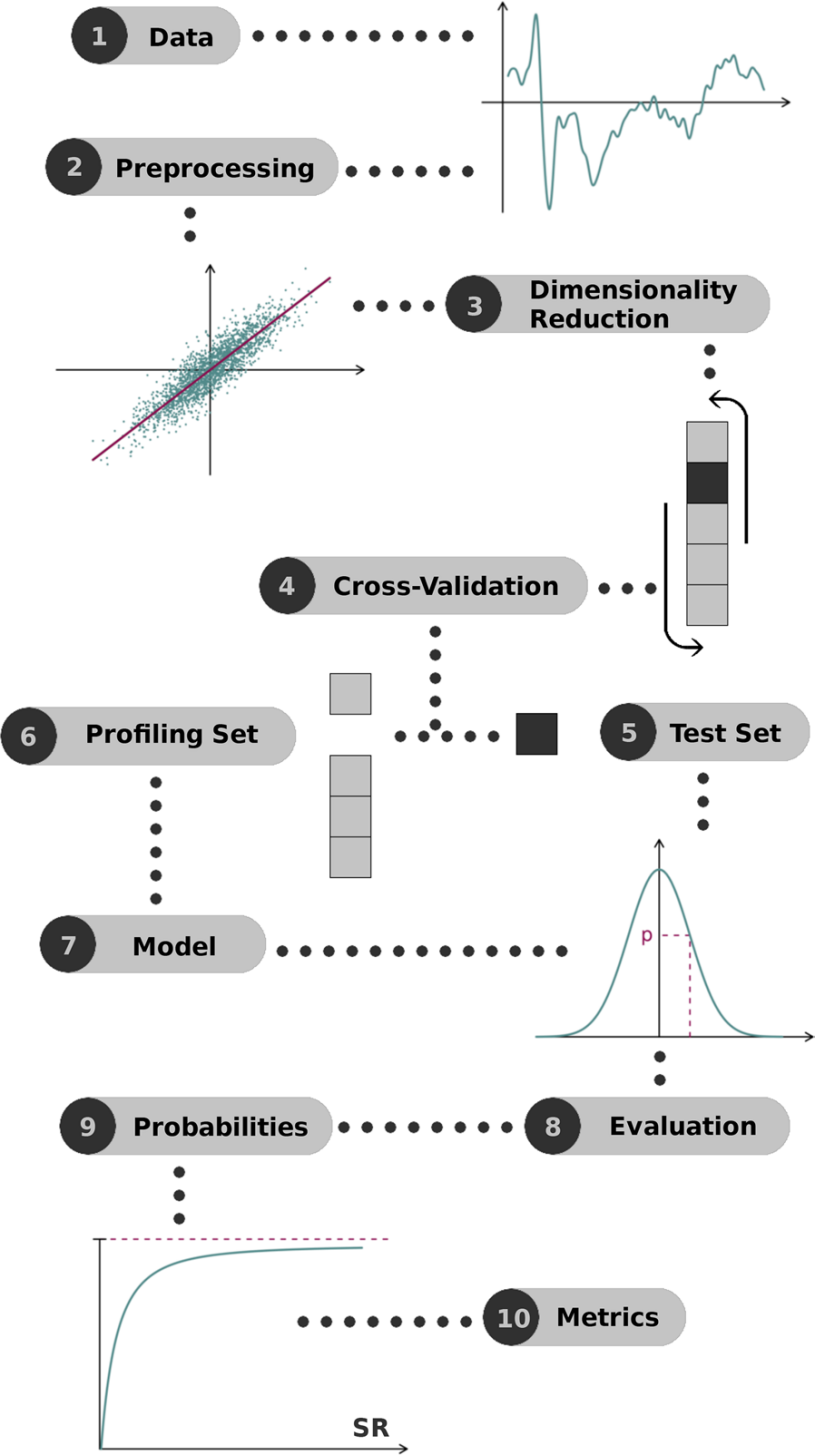
Evaluation methodology

The scheme of Fig. 1 represents the general procedure we followed to analyze our EEG data (similar to side-channel analysis). We next detail its main steps.

*Preprocessing* As a first step, all the observations were preprocessed using a bandpass filter. We set the low-frequency cutoff to 0.5 Hz to remove the slow drifts in the EEG signals and the high-frequency cutoff to 30 Hz to remove muscle artifacts and 50 Hz noise.

**Fig. 2:**
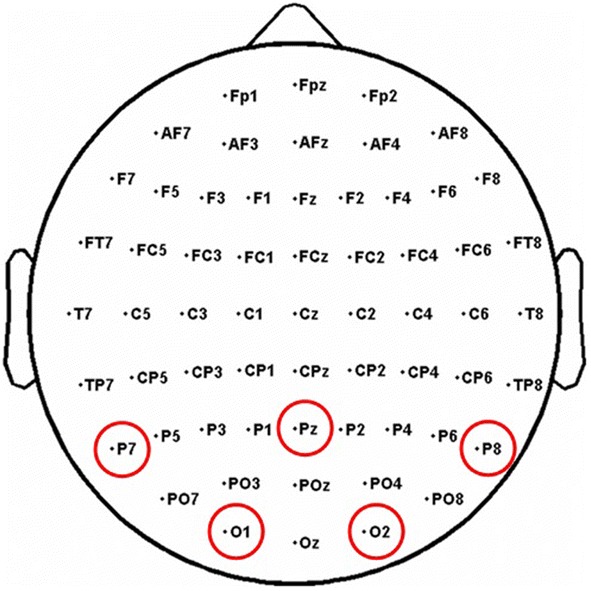
Repartition of the electrodes on the scalp

*Selection of electrodes* As mentioned in introduction, each original observation is made of 32 vectors of 1000 samples, leading to a large amount of data to process. To simplify our treatments, we started by analyzing the different electrodes independently. Among the 32 ones of our cap, Electrodes P7, P8, Pz, O1 and O2 gave rise to non-negligible signal (see Fig. 2), which is consistent with the existing literature where ERPs related to semantic associations and incongruities were exhibited in the central/parietal zones [10–12]. Our following analyses are based on the exploitation of the Electrodes P7 and P8 which provided the most regular information across the different users.^2^

**Fig. 3:**
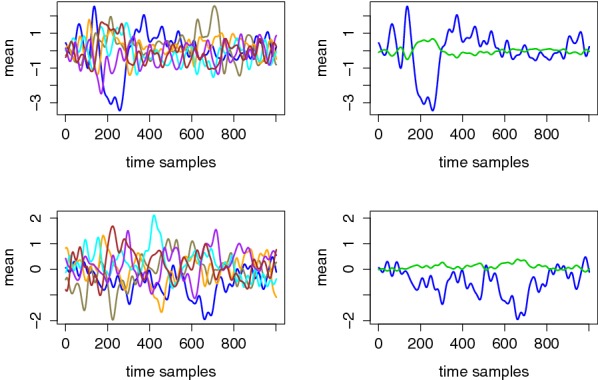
Exemplary mean traces for different tag (left) and PIN (right) values. Top: User 8, Electrode P7. Bottom: User 6, Electrode P7

**Fig. 4:**
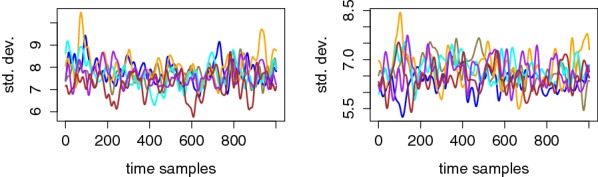
Exemplary standard deviation traces for different tag values corresponding to User 8, Electrode P7 (left) and User 6, Electrode P7 (right)

For illustration, Figs. 3 and 4 represent the mean and standard deviation traces corresponding to two different users. (Similar figures for the other users are available in appendices, as shown in Figs. 13, 14 and Figs. 15, 16.) From these examples, a couple of relevant observations can already be extracted (and will be useful for the design and interpretation of our following evaluations). First, we see (on the left parts of Fig. 3) that the EEG signals may be more or less informative depending on the users and electrodes. More precisely, we generally noticed informative ERP components after 300–600 ms (known as the P300 component) for most users and electrodes, which is again consistent with the existing literature [10–12]. Yet, our measurements also put forward user-specific differences in the shape of the mean traces corresponding to the correct PIN value. (Note that the figures mostly show examples of informative EEG signals, but for one user and some other electrodes, no such clear patterns appear.) Second, and quite importantly, the difference between the left and right parts of the figures illustrates the significant gain when moving from an unsupervised/unprofiled evaluation context to a supervised/profiled one. That is, while in the first case, we need the traces corresponding to the correct PIN value to stand out, in the second case, we only need it to behave differently than the others.

Eventually, a look at the standard deviation curves in Fig. 4 suggests that the measurements are quite noisy, hence non-trivial to exploit with a limited amount of observations. This will be confirmed in our following PDF estimation phase and therefore motivates the dimensionality reduction in the next section (intuitively because using more dimensions can possibly lead to better signal extraction, which can mitigate the effect of a large noise level).

*Dimensionality reduction* The evaluation of our metrics requires to build a probabilistic model, which may become data intensive as the number of dimensions in the observations increases. For example, directly estimating a 2000-dimensional PDF corresponding to our selected electrodes is not possible. In order to deal with this problem, we follow the standard approach of reducing dimensionality. More precisely, we use the principal component analysis (PCA) that was shown to provide excellent results in the context of side-channel attacks against cryptographic devices [19]. We investigate two options in this direction.

**Fig. 5:**
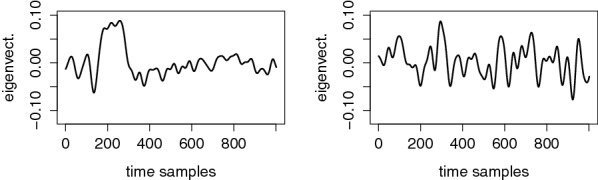
Exemplary eigenvectors for the average PCA, corresponding to User 8, Electrode P7. Left: first dimension. Right: fifth dimension

**Fig. 6:**
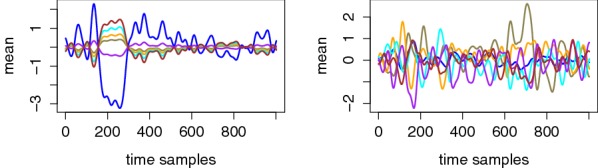
Reconstructed signal based on average PCA, corresponding to User 8, Electrode P7, using the first dimension (left) and all the other dimensions (right)

First, and looking at the observations in Fig. 3, it appears that the mean traces corresponding to the different tags are quite discriminant regarding the value of *p*. Hence, and as in [19], a natural option is to compute the projection vectors of the PCA based on these mean traces. This implies computing average vectors $\bar{\varvec{o}}^j={\mathsf {E}}_{i\approx 1}^{150} \varvec{o}_i^j$, and then to derive the PCA eigenvectors based on the $\bar{\varvec{o}}^j$’s, which we denote as $\varvec{R}_{1:N_d}\leftarrow {\mathsf {PCA}}\big (\{\bar{\varvec{o}}^j\}_{j=1:6}\big )$, where $N_d$ is the number of dimensions to extract. Due to the limited number of mean traces (i.e., 6), we can only compute $N_d=5$ eigenvectors and therefore are limited to five-dimensional attacks in this case.^3^ However, it turned out that in our experiments, this version of the PCA extracts most of the relevant samples in the first dimension. This is intuitively witnessed by Fig. 5 which represents the first and fifth eigenvectors corresponding to User 8 and Electrode P7 (i.e., $\varvec{R}_{1}$ and $\varvec{R}_{5}$): we indeed observe that the first dimension corresponds to the points of interest in Fig. 3, while the fifth one seems to be dominated by noise. In the following, we will denote this solution as the “average PCA”. Note that such a dimensionality reduction does not take advantage of any secret information (i.e., it is not a supervised/profiled one) since it builds the mean traces based on public tags. In order to further confirm that the first dimension of the average PCA extracts relevant information from our observations, Fig. 6 additionally illustrates reconstructed signals for this first and all the other dimensions.

Yet, one possible drawback of the previous method is that estimating the average traces $\bar{\varvec{o}}^j$ becomes expensive when the number of PIN codes increases. In order to deal with and quantify the impact of this limitation, we also considered a “raw PCA,” where we directly reduce the dimensionality based on raw traces, next denoted as $\varvec{R}_{1:N_d}\leftarrow {\mathsf {PCA}}\big (\{\varvec{o}_i\}_{i\approx 1:900}\big )$. While this approach is not expected to extract the information as effectively, it allows deriving a much larger number of dimensions than in the previous (average) case. Concretely though, exploiting dimensions 1–5 only was a good trade-off between the informativeness of the dimensionality reduction, the risk of overfitting (useless) dataset-dependent patterns and the risk of outliers in our experiments (see the paragraph on outliers).

As a result of this dimensionality reduction phase, the observation vectors $\varvec{o}(1$:2000) (which correspond to the concatenation of the measurements for our two selected electrodes) are reduced to smaller vectors $\varvec{R}_{1:N_d}\times \varvec{o}$ (i.e., each dimension *o*(*d*) corresponds to the scalar product between the original observations $\varvec{o}$ and a 2000-element vector $\varvec{R}_d$). We recall that PCA is not claimed to be an optimal dimensionality reduction, since it optimizes a criteria (i.e., the variance between the raw or mean traces) which does not capture all the information in our measurements. However, it is a natural first step in our investigations, and we could verify that our following conclusions are not affected by slight variations of the number of extracted dimensions (i.e., adding one or two dimensions), which therefore fits our (primary) confidence and stability goal.

*PDF estimation* We now describe the main ingredient of our supervised/profiled evaluation, namely the PDF estimation for which we exploit the knowledge of the *p* values for the observations in the profiling sets.

In order to build a model $\hat{\mathsf {f}}_{\mathrm {model}}(\varvec{o}_{1:N_d}|p)$, we first take advantage of the fact that the dimensions of the $\varvec{o}_{1:N_d}$ vectors after PCA are orthogonal. By additionally considering them as independent, this allows us to reduce the PDF estimation problem from one $N_d$-variate one to $N_d$ univariate ones. Based on this simplification, the standard approach in side-channel analysis is to assume the observations to be normally distributed and to build Gaussian templates [20]. Yet, in our experiments no such obvious assumption on the distributions in hand was a priori available. As a result, we first considered a (nonparametric) kernel density estimation as used in [21], which has slower convergence but avoids any risk of biased evaluations [22]. Kernel density estimation is a generalization of histograms. Instead of bundling samples together in bins, it adds (for each observation) a small kernel centered on the value of the observation to the estimated PDF. The resulting estimation that is a sum of kernels is smoother than histograms and usually converges faster. Concretely, kernel density estimation requires selecting a kernel function (we used a Gaussian one) and to set the bandwidth parameter (which can be seen as a counterpart to the bin size in histograms). The optimal choice of the bandwidth depends on the distribution of the observations, which is unknown in our case. So we need to rely on a heuristic and used Silverman’s rule-of-thumb for this purpose [23].^4^

*Evaluation metrics* Following the general principles put forward in [17], our evaluations will be based on a combination of information-theoretic and security analyses. The first ones aim at evaluating whether exploitable information is available in the EEG signals; the second ones at evaluating how efficiently this information can be exploited to mount a side-channel attack. Note that since we do not assume the users to behave identically, these metrics will always be evaluated and discussed for each user independently.

*Perceived information* The perceived information (PI) was introduced in the context of side-channel attacks against cryptographic devices, of which the goal is to recover some secret data (aka key) given some physical leakage [24]. The PI aims at quantifying the amount of information about the secret key, independent of the adversary who will exploit this information. Informally, we will use this metric in a similar way, by just considering *P* as a bit to recover and the observations as leakages. Using the previous notations, we define the PI between the PIN random variable *P* and the observation random variable $\varvec{O}$:

$$\begin{aligned} {\mathrm {PI}}(P;\varvec{O})={\mathrm {H}}[P]+\sum _{p}\Pr [p]\cdot \int _{\varvec{o}} {\mathsf {f}}(\varvec{o}|p) \cdot \log _2 \Pr _{\mathrm {model}}[p|\varvec{o}] \mathrm{d}\varvec{o}, \end{aligned}$$

where we use the notation $\Pr [X=x]=:\Pr [x]$ for conciseness, and ${\mathsf {f}}(\varvec{o}|p)$ is the (continuous) PDF of the observations given the value of *p*. In the ideal case where the model is perfect, the PI is identical to Shannon’s mutual information. In the practical cases where the model differs from the observation’s true distribution, the PI captures the amount of information that is extracted from these observations, biased by the model (assumption and estimation) errors [22].

Of course, concretely the true distribution ${\mathsf {f}}(\varvec{o}|p)$ is unknown to the adversary/evaluator and can only be sampled. Therefore, the approach in side-channel analysis, that we repeat here, is to split the set of observations $\mathcal {O}$ in *k* non-overlapping sets ${\mathcal {O}}^{(i)}$. We then define the profiling sets ${\mathcal {O}}_{\mathsf {p}}^{(j)}=\bigcup _{i\ne j} {\mathcal {O}}^{(i)}$ and the test sets ${\mathcal {O}}_{\mathsf {t}}^{(j)}={\mathcal {O}} {\setminus } {\mathcal {O}}_{\mathsf {p}}^{(j)}$. The PI is computed in two phases:

1. The observations’ conditional distribution is estimated from a profiling set. We denote this phase with
  $$\begin{aligned} \hat{\mathsf {f}}^{(j)}_{\mathrm {model}}(\varvec{o}|p)\leftarrow {\mathcal {O}}_{\mathsf {p}}^{(j)}. \end{aligned}$$
  Note that the $\Pr _{\mathrm {model}}[p|\varvec{o}]$ factor involved in the PI definition is directly derived via Bayes’ theorem as:
  $$\begin{aligned} \hat{\Pr }_{\mathrm {model}}[p|\varvec{o}]=\frac{\hat{\mathsf {f}}^{(j)}_{\mathrm {model}}(\varvec{o}|p)\cdot \Pr [p]}{\sum _{p^*} \hat{\mathsf {f}}^{(j)}_{\mathrm {model}}(\varvec{o}|p^*)\cdot \Pr [p^*]}\cdot \end{aligned}$$
2. The model is tested by computing the PI estimate:
  $$\begin{aligned}\hat{\mathrm {PI}}^{(j)}(P;\varvec{O})={\mathrm {H}}[P]+\sum _{p=0}^1\Pr [p]\cdot \sum _{\varvec{o}\in {\mathcal {O}}_{\mathsf {t}}^{(j)}|p} \frac{1}{n_{p}^j} \cdot {\log} _{2} \hat{\mathrm{Pr}}_{\mathsf{model}}[p|\varvec{o}], \end{aligned}$$
  with $n_{p}^j$ the number of observations in the test set ${\mathcal {O}}_{\mathsf {t}}^{(j)}|p$.

Eventually, the *k* outputs $\hat{\mathrm {PI}}^{(j)}(P;\varvec{O})$ are averaged to get an unbiased estimate, and their spread characterizes the accuracy of the result. Note that concretely, the maximum size for the profiling set in our experiments equals $\approx 899$, leading to a cross-validation parameter $k\approx 900$ and a test set of size 1. In this case, the model building phase is repeated $\approx 900$ times, and each model is tested once against an independent sample. (We use the $\approx$ symbol to reflect the fact that these values are approximated, due to the rejection of eye blinks mentioned in Sect. 2.) This “leave one out” strategy has a large cross-validation parameter compared to current practice (e.g., in side-channel attacks against cryptographic implementations a value of $k=10$ was selected [22]), leading to computationally intensive evaluations. Yet, it is justified in our study because of the limited number of samples available in our experiments.

*Success rate and average rank* In order to confirm that the estimated PI indeed leads to concrete attacks, we consider two simple security metrics. Here, the main challenge is that we only have models for the real and random PIN codes, while the actual observations in the test set naturally come from six different events. As a result, we first considered the success rate event per event. For this purpose, the $\approx 900$ observations are split in 6 sets of $\approx 150$ observations that correspond to the six different tag values. Based on these 6 sets, we can compute the probability that the observations are correctly classified as real or random in function of the number of observations exploited in the attack, next denoted as *q*. This is done by averaging a success function $\mathsf {S}$ that is computed as follows. If $q=1$: $\mathsf {S}(\varvec{o}_1)=1$ if $\hat{\Pr }_{\mathsf {model}}[p|\varvec{o}_1]>\hat{\Pr }_{\mathsf {model}}[\bar{p}|\varvec{o}_1]$ and $\mathsf {S}(\varvec{o}_1)=0$ otherwise (where $\bar{p}$ denotes the incorrect event); if $q=2$: $\mathsf {S}(\varvec{o}_1,\varvec{o}_2)=1$ if $\hat{\Pr }_{\mathsf {model}}[p|\varvec{o}_1]\times \hat{\Pr }_{\mathsf {model}}[p|\varvec{o}_2]>\hat{\Pr }_{\mathsf {model}}[\bar{p}|\varvec{o}_1]\times \hat{\Pr }_{\mathsf {model}}[\bar{p}|\varvec{o}_2];\ldots$ Concretely, this success rate is an interesting metric to check whether the observations generated by different incorrect PIN values indeed behave similarly.

Of course, an adversary eventually wants to compare the likelihoods of different PIN values. For this purpose, we also considered the average rank of the correct PIN in an experiment where we gradually increase the number of observations per tag *q*, but this time consider sets of 6 observations at once that we classify only according to the model for the real PIN. This leads to vectors $(\hat{\Pr }_{\mathrm {model}}[p|\varvec{o}_1^1],\hat{\Pr }_{\mathrm {model}}[p|\varvec{o}_1^2],\hat{\Pr }_{\mathrm {model}}[p|\varvec{o}_1^3],\ldots,$ $\hat{\Pr }_{\mathrm {model}}[p|\varvec{o}_1^6])$ if $q=1$, $(\hat{\Pr }_{\mathrm {model}}[p|\varvec{o}_1^1] \times$ $\hat{\Pr }_{\mathrm {model}}[p|\varvec{o}_2^1],$ ..., $\hat{\Pr }_{\mathrm {model}}[p|\varvec{o}_1^6]\times \hat{\Pr }_{\mathrm {model}}[p|\varvec{o}_2^6])$ if $q=2$, ..., where the superscripts denote the tag from which the observations originate. The average rank is then obtained by sorting this vector and estimating the sample mean of the position of the tag 1 in the sorted vector.

*Connecting the metrics (sanity check)* Note that as discussed in [25], information-theoretic and security metrics can be connected (i.e., a model that leads to a positive PI should lead to successful attacks).^5^ We consider both types of metrics in our experiments because the first ones allow a better assessment of the confidence in the evaluations (see the following paragraph on confidence), while the second ones lead to simpler intuitions regarding the concrete impact of the attacks.

*Outliers* As mentioned in the Dimensionality Reduction paragraph, the main drawback of the raw PCA is that it extracts the useful EEG information less efficiently, which we mitigate by using more dimensions. Unfortunately, this comes with an additional caveat. Namely, the less informative information extraction combined with the addition of more dimensions increases the risk of outliers (i.e., observations that would classify the correct PIN value very badly for some dimensions, possibly leading to a negative PI). In this particular case, we considered an additional post-processing (after the dimensionality reduction and model building phases). Namely, given the $\approx 900$ probabilities $\hat{\Pr }[p|\varvec{R}_{1:N_d}\times \varvec{o}_i]$, we rejected the ones below 0.001 and set them to this minimum value. This choice is admittedly heuristic, yet did consistently lead to positive results for all the users. It is motivated by limiting the weight of the log probabilities for the outliers in the PI estimation. We insist that this treatment of outliers is only needed for the raw PCA. For the average PCA, we did not reject any observation (other than the ones in Sect. 2).

*Confidence* By using $\approx 900$-fold cross-validation, we can guarantee that our PI estimates will be based on 900 observations, leading to 900 values for the log probabilities $\log _2(\hat{\Pr }[p|\varvec{R}_{1:N_d}\times \varvec{o}_i])$. Since this remains a limited amount of data compared to the case of side-channel attacks against cryptographic implementations, and the extracted PI values are small, we completed our information-theoretic evaluations by computing a confidence interval for the PI estimates. To avoid any distribution-specific assumption, we computed a 10% bootstrap confidence interval [26], by resampling 100 bootstrap samples out of our 900 log probabilities, computing 100 mean bootstrap samples, sorting them and using the 95th and 5th percentiles as the endpoints of the intervals.^6^ For simplicity, this was only done for the PI metric and not for the success rate and average rank since (1) successful Bayesian attacks are implied by the information-theoretic analysis [25], (2) these metrics are more expensive to sample (e.g., we have only one evaluation of the success function with $q\approx 150$ per user), and (3) they are only exhibited to provide intuitions regarding the exploitability of the observations (i.e., the attack complexities).

### Unsupervised (aka non-profiled) analysis

While supervised (aka profiled) analyses are the method of choice to gain understanding about the information available in a side-channel, their practical applicability is of course questionable. Indeed, building a model for a target user may not always be feasible, and this is particularly true in the context of attacks against the human brain since, as will be discussed in Sect. 4.3, models built for one user are not always (directly) exploitable against another user. In this section, we therefore propose an unsupervised/non-profiled extension of the previous (supervised/profiled) information-theoretic evaluation. To the best of our knowledge, this variation was never described as such in the open literature (although it shares some similarities with the non-profiled attacks surveyed in [21]). For this purpose, our starting point is the observation from Fig. 3, that in an unsupervised/non-profiled context, one can take advantage of the fact that the (e.g., mean) traces of the EEG signals corresponding to the correct PIN value may stand out. As a result, a natural idea is to compute the PI metric 6 times independently, each time assuming a different (possibly random) tag to be correct during an “on-the-fly” modeling phase. If the traces corresponding to the (truly) correct PIN are more singular (comparatively to the others), we can expect the PI estimated with this PIN to be larger, leading to a successful attack.

Of course, such an attack implies an additional neurophysiological assumption (while in the supervised/profiled setting, we just exploit any information available). Yet, it nicely fits the intuitions discussed in the rest of this section, which makes it a good candidate for concrete evaluation. Furthermore, we mention that directly recovering the correct PIN value may not always be necessary: as in the case of side-channel analysis, reducing the rank of the correct PIN value down to an enumerable one may be sufficient [18].

## Experimental results

### Supervised (aka profiled) evaluation

As in the previous section, we start with the results of our supervised/profiled evaluations, which will be in two (information-theoretic and security) parts. Beforehand, there is one last choice regarding the computation of $\hat{\Pr }[p|\varvec{R}_{1:N_d}\times \varvec{o}_i]$ via Bayes’ theorem. Namely, should we consider maximum likelihood or maximum a posteriori attacks (i.e., should we take advantage of the a priori knowledge of $\Pr [p]$ or consider a uniform a priori). Interestingly, in our context ignoring this a priori and performing maximum likelihood attacks is more relevant, since we mostly want to avoid false negatives (i.e., correct PINs that would be classified as random ones), which prevent efficient enumeration. Since the a priori on *P* increases the amount of such errors (due to the a priori bias of 5/6 toward random PIN values), the rest of this section reports on the results of maximum likelihood attacks.

#### Perceived information

**Fig. 7:**
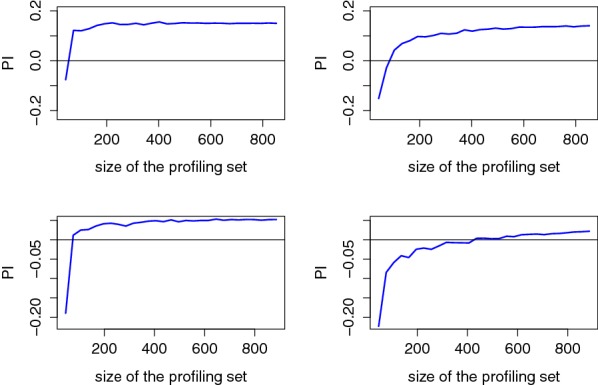
Evolution of the PI in function of the size of the profiling set for Users 3 (top) and 6 (bottom), using average PCA (left) and raw PCA (right)

As a first step in our evaluations, we estimated the PI using the methodology described in the previous section. We started by looking at the evolution of the PI estimation in function of the number of observations in the profiling set used to build the model. The results of this analysis for a couple of users are in Fig. 7 (Fig. 17 in appendix contains the results for all users) from which two quantities must be observed:

- The value of the PI estimated using the maximum profiling set (i.e., the extreme right values in the graphs). It reflects the informativeness of the model built in the profiling phases and is correlated with the success rate of the online (maximum likelihood) attack using this model [25]. Positive PI values indicate that the model is sound (up to Footnote 5) and should lead to successful online attacks if the number of observations (i.e., the *q* parameter in our notations) used by the adversary is sufficient.
- The number of traces in the profiling set required to reach a positive PI. It reflects the (offline) complexity of the model estimation (profiling) phase [27].

In this respect, the results in Fig. 7 show a positive convergence for the two illustrated users, yet toward different PI values which indicate that the informativeness of the EEG signals differs between them. Next, and quite interestingly, we also see that the difference between average PCA (in the left part of the figure) and raw PCA (in the right side) confirms the expected intuitions. Namely, the fact that raw PCA reduces dimensionality based on less meaningful criteria and requires more dimensions implies a slower model convergence. Typically, model convergence was observed in the 100 observations’ range with average PCA and required up to 400 traces with raw PCA. For completeness, Table 1 contains the estimated PI values with maximum profiling set, for the different users and types of PCA. Excepted for one user (User 5) for which we could never reach a positive PI value with confidence,^7^ this analysis suggests that all the users lead to exploitable information and confirms the advantage of average PCA. A similar table obtained with the Gaussian profiling is given in Appendix 1.

Note that we leave the accurate treatment of confidence intervals for Sect. 4.2 where it will play an important role. Yet, we can already notice the stable shape of the PI curves as the size of the profiling set increases, which intuitively indicates the convergence of our estimations.

**Table 1:** Estimated PI values with maximum profiling set

User	$\hat{\mathrm {PI}}(P;O)$ with avg. PCA	$\hat{\mathrm {PI}}(P;O)$ with raw PCA
1	0.0739	0.0618
2	0.1643	0.1315
3	0.1494	0.1398
4	0.0920	0.0228
5	$\varnothing$	$\varnothing$
6	0.0521	0.0214
7	0.0759	0.0568
8	0.1697	0.0458

#### Success rate and average rank

As discussed in the previous section, our information-theoretic analysis is a method of choice to determine whether discriminant information can be extracted from EEG signals with confidence. Yet, it does not lead to obvious intuitions regarding the actual complexity of an online attack where an adversary obtains a set of *q* fresh observations and tries to detect whether some of them correspond to a real PIN value. Therefore, we now provide the results of our complementary security analysis and estimate the success rate and average key rank metrics. As previously mentioned these evaluations are less confident, since for large *q* values such as $q=150$ we can have only one evaluation of the success function. Concretely, the best success rate/average key rank estimates are therefore obtained for $q=1$. We took advantage of resampling when estimating them for larger *q*’s.

**Fig. 8:**
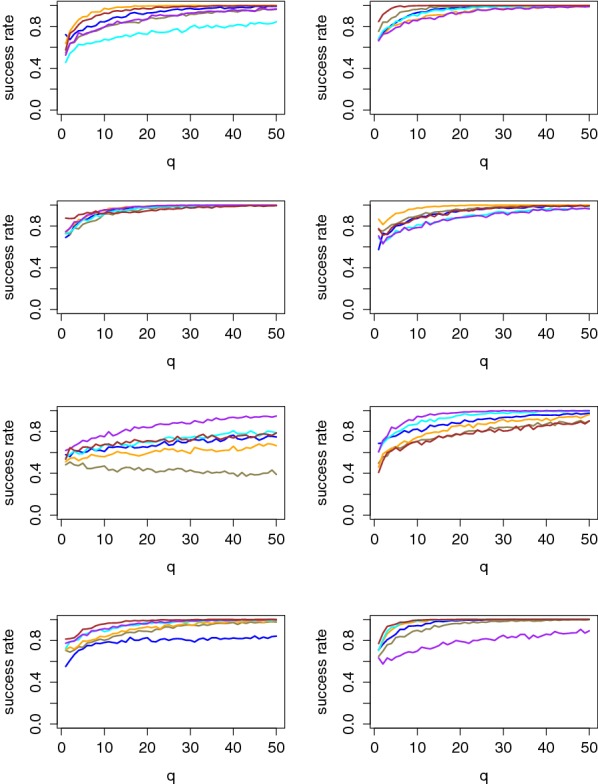
Success rates per tag value for Users 1, 3, 5 and 7 (left column) and Users 2, 4, 6 and 8 (right column)

**Fig. 9:**
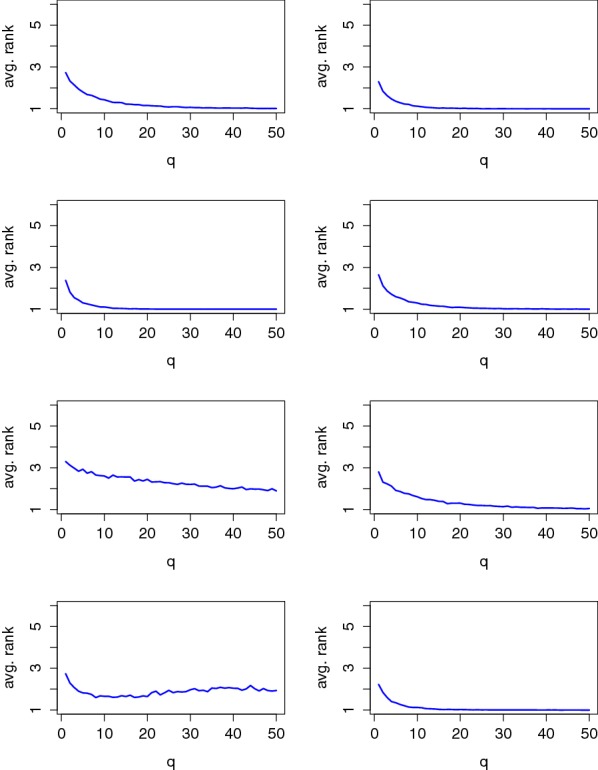
Average rank of the correct PIN for Users 1, 3, 5 and 7 (left column) and Users 2, 4, 6 and 8 (right column)

Figures 8 and 9 illustrate that these metrics are indeed correlated with the value of the PI estimates using the maximum profiling set, which explains the more efficient attacks against Users 2, 3 and 8. Concretely, the average rank figure suggests that correct PIN value can be exactly extracted in our 6-PIN case study with 5–10 observations for the most informative users and 30–40 observations for the least informative ones. The success rate curves also bring meaningful intuitions since they highlight that all (correct and random) PIN values can be correctly classified with our profiled models (in slightly more traces). This confirms our neurophysiological assumption from the previous section that the users react similarly to all random values.^8^

Besides, Fig. 8 is interesting since it shows how confidently the correct PIN value is classified independent of the others. Hence, its results would essentially scale with larger number of PIN values.

Finally, Fig. 9 confirms the presence of a parasitic familiar event for User 5, for which the average rank is reduced to 2 rather than optimal 1.^9^

### Unsupervised (aka non-profiled) analysis

**Fig. 10:**
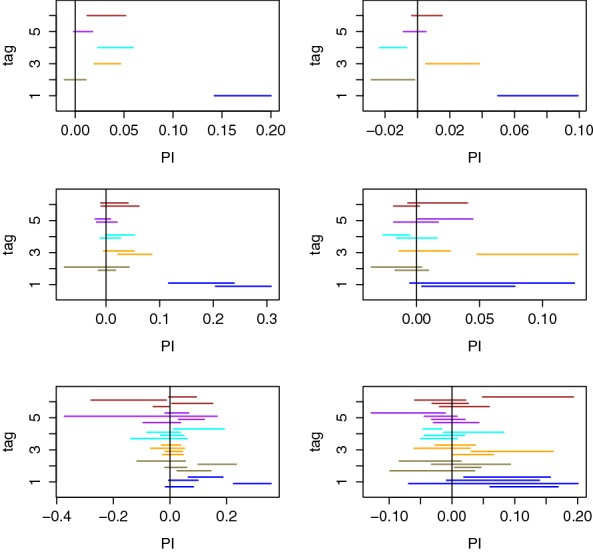
Confidence intervals for the (non-profiled) PI evaluation of Sect. 3.3 with $\approx 900$ observations (top), $\approx 450$ observations (middle) and $\approx 225$ observations (bottom), for Users 8 (left) and 6 (right)

We now move to the more challenging problem of unsupervised/non-profiled attacks. For this purpose, we first applied the attack sketched in Sect. 3.3 with the maximum number of traces in the profiling set. That is, we repeated our evaluation of the PI metric six times, assuming each of the tag values to be the real one. Furthermore, we computed the confidence intervals for each of the PI estimates according to the confidence paragraph in the previous section. The results of this experiment are in Fig. 10 for two users and lead to three observations.

First, looking at the first line of the figure, which corresponds to the correct PIN value, we can now confirm that the PI estimates of Sect. 4.1.1 are sufficiently accurate (e.g., the confidence intervals clearly guarantee a positive PI). Second, the confidence intervals for the random PIN values (i.e., tags 2–6) confirm the observation from our success rate curves (Fig. 8) that the users react similarly to all random values. Third, the middle and bottom parts of the figure show the results of two (resp. 4) non-profiled attacks where the profiling set was split in 2 (resp. 4) independent parts (without resampling), therefore leading to the evaluation of 2 (resp. 4) confidence intervals for each tag value. Concretely, the PI estimate for the correct PIN value consistently started to overlap with the ones of random PINs for all users, as soon as the number of attack traces *q* was below 200, and no clear gain for the correct PIN could be noticed below $q=100$. This confirms the intuition that unsupervised/non-profiled side-channel attacks are generally more challenging than supervised/profiled ones (here, by an approximate factor 5–10 depending on the users).

This conclusion also nicely matches the one in Sect. 4.1.1, Fig. 7, where we already observed that the (offline) estimation of an informative model is more expensive than its (online) exploitation for PIN code recovery as measured by the success rate and average rank (by similar factors). Indeed, in the unsupervised/non-profiled context such an estimation has to be performed “on-the-fly”.

### Model portability

Since the previous section suggests a significant advantage of supervised/profiled attacks over unsupervised/non-profiled ones, a natural question is whether the profiling can lead to realistic attack models. Clearly, estimating a model for the correct PIN of each user an adversary would like to target seems hardly realistic (especially if 10,000 PIN values are considered). Therefore, and in order to get around this drawback, a solution would be to use the model built for one user against another user. Despite limited by the number of users in our experiments, we made preliminary analyses in this direction. Interestingly, while for most pairs of users the resulting attacks failed and the PI estimates remained negative, we also found two pairs of users for which the models could be mutually exchanged. Namely, targeting User 1 (resp. User 6) with the model of User 6 (resp. User 1) leads to a PI of 0.0211 (resp. 0.0357). And targeting User 1 (resp. User 3) with the model of User 3 (resp. User 1) leads to a PI of 0.0281 (resp. 0.0246). Intuitively, this positive result is in part explained by the similar shapes of the first eigenvectors used to reduce the dimensionality when estimating these models. Overall, this problem of model portability is in fact similar to the problem of variability faced in the context of side-channel attacks against cryptographic devices [24]. Hence, it is an interesting scope for further research to investigate how advanced profiling techniques (e.g., profiling multiple users jointly with mixture models) could be used to increase the practical relevance of supervised/profiled attacks against the human brain.

**Fig. 11:**
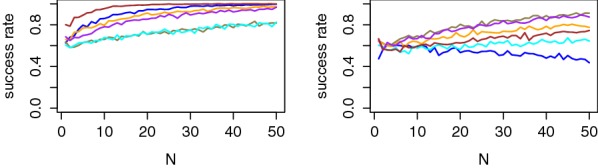
Exemplary success rates per tag value for “all against one” profiling: (left: User 3, right: User 4)

Note that in this context, the impact of certain parameters in our methodology is susceptible to evolve too. For example, and as just mentioned, the user specificities that make the portability of the models challenging are in part due to the shape of the eigenvectors produced by the average PCA. So using the raw PCA may gain interest in this case. As a preliminary experiment in this direction, we evaluated the PI when targeting a user with a model profiled with all the other users.^10^ As a result, we could obtain positive PI values for 5 out of 7 users, with both the average and the raw PCA (and similar informativeness). For illustration, the success rate curves for such a (successful and unsuccessful) profiling are given in Fig. 11. These results suggest that profiling classes of similar users is certainly a promising approach for realistic attacks.

## From security issues to privacy issues

**Fig. 12:**
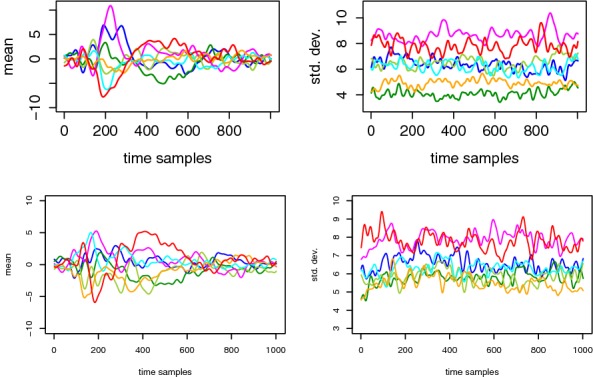
Exemplary mean traces (left) and standard deviation traces (right) for the reaction of different users to the correct PIN value (Electrode P8 on top row and Electrode P7 on bottom row)

**Fig. 13:**
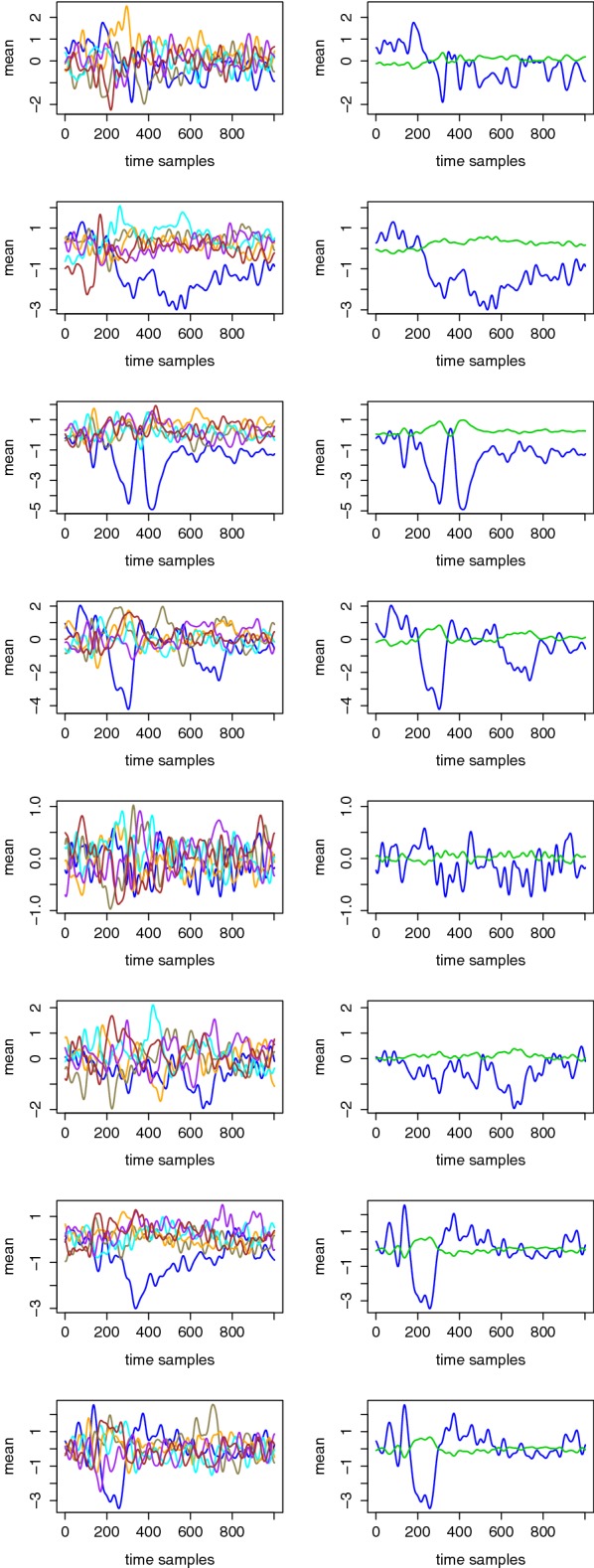
Mean traces for different tag (left) and PIN (right) values. User 1 (top) to 8 (bottom), Electrode P7

**Fig. 14:**
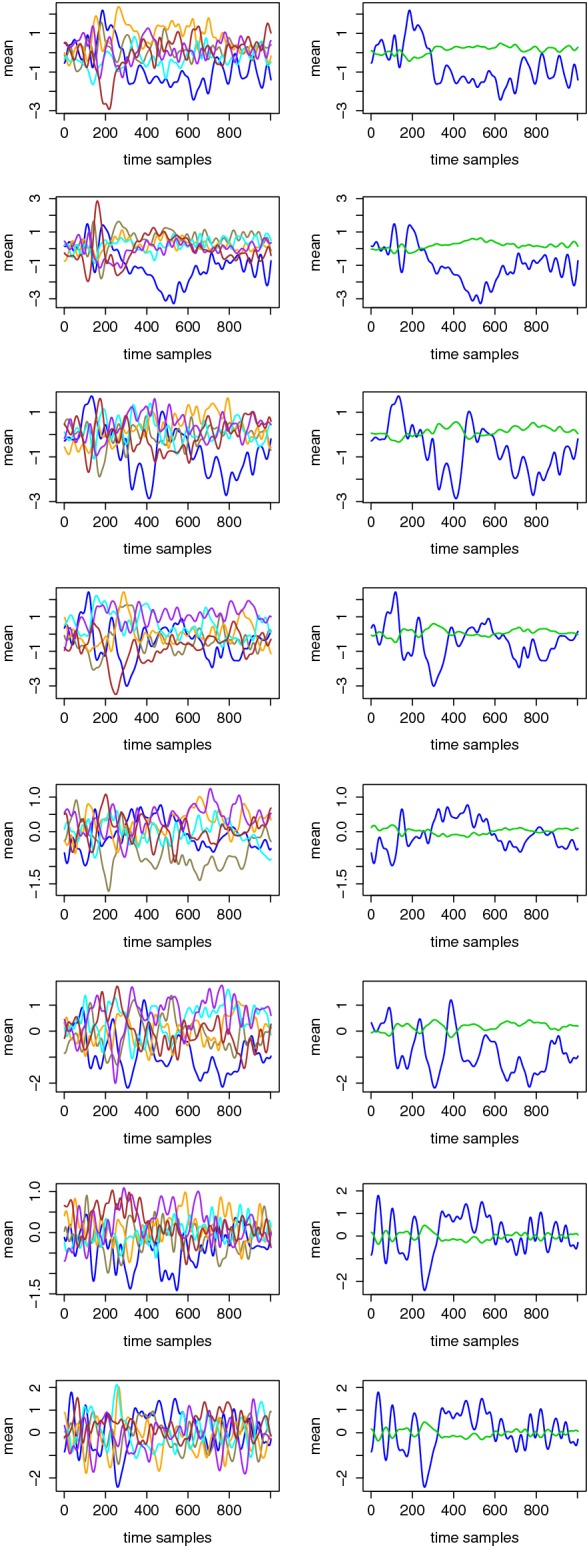
Mean traces for different tag (left) and PIN (right) values. User 1 (top) to 8 (bottom), Electrode P8

**Fig. 15:**
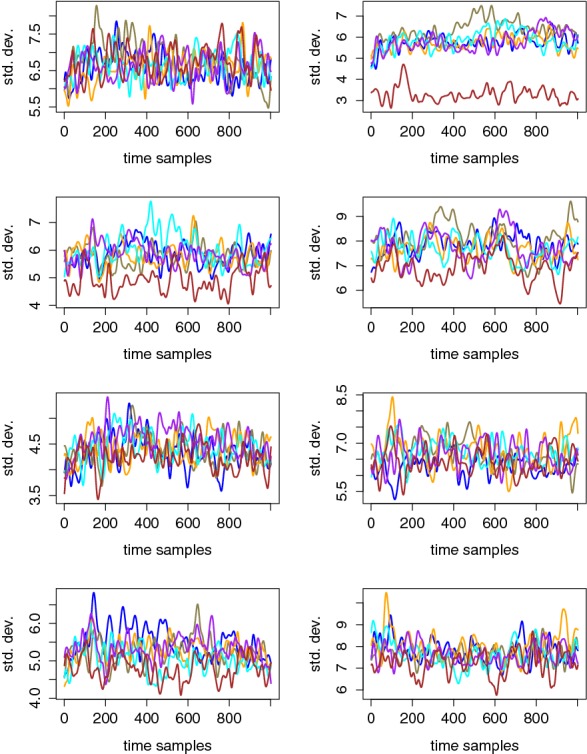
Standard deviation traces for different tag values corresponding to Users 1, 3, 5 and 7 (left) and Users 2, 4, 6 and 8 (right), Electrode P7

**Fig. 16:**
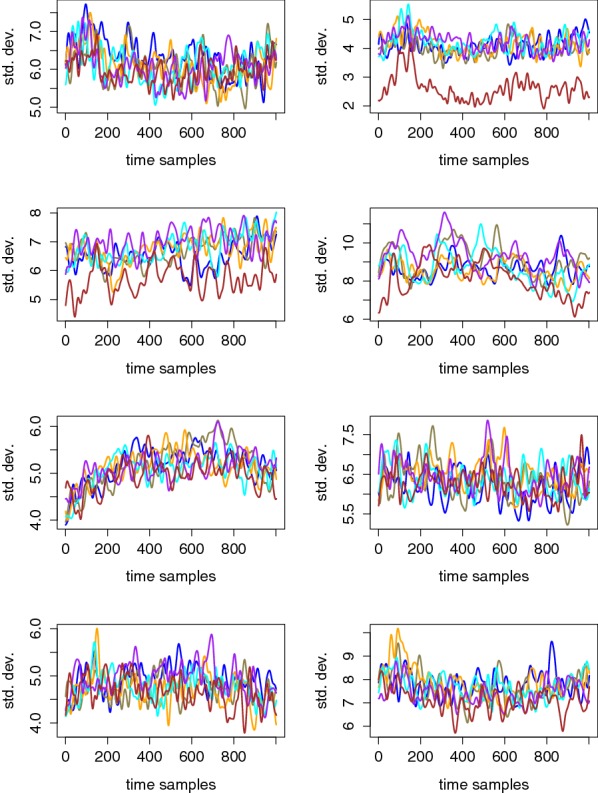
Standard deviation traces for different tag values corresponding to Users 1, 3, 5 and 7 (left) and Users 2, 4, 6 and 8 (right), Electrode P8

**Fig. 17:**
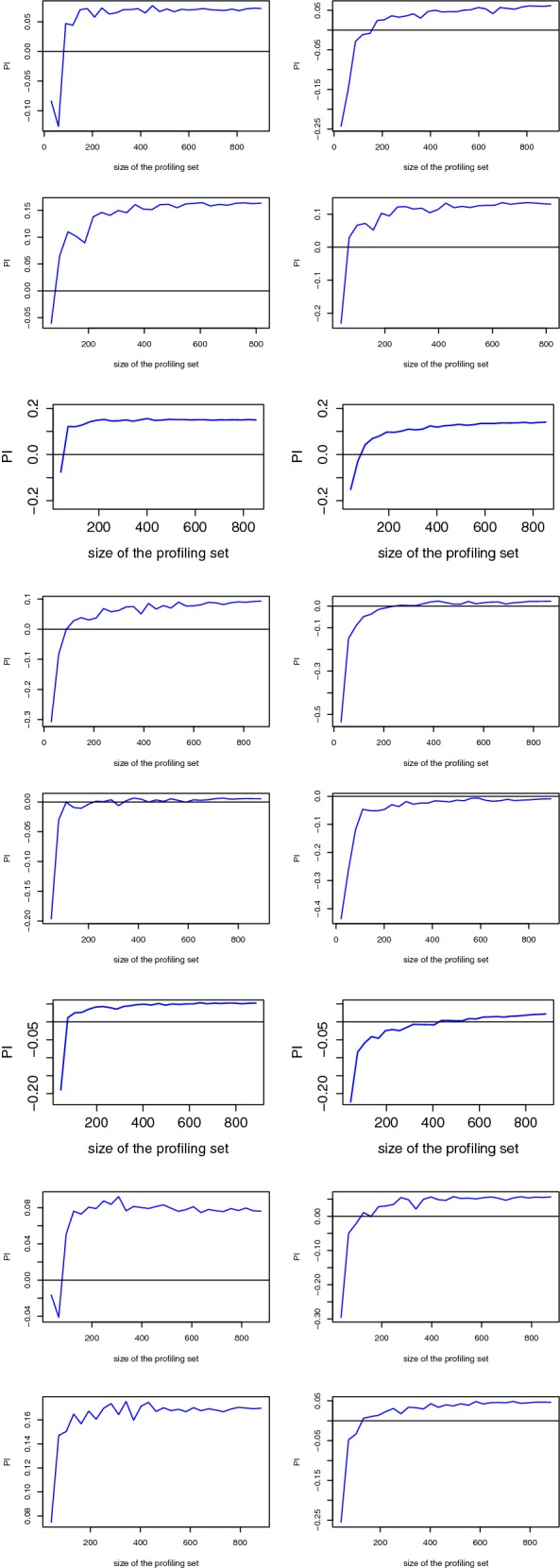
Evolution of the PI in function of the size of the profiling set for Users 1 (top) to User 8 (bottom), using average PCA (left) and raw PCA (right)

Before concluding, we make a short excursion from the evaluation of security toward the risks of privacy in BCI-based applications. That is, since the previous investigations exhibited significant differences between the EEG signals of different users reacting to their correct PIN values, we reverse the problem and now try to identify the users rather than the PIN values. For this purpose, we followed exactly the same methodology and estimated the modified perceived information $\hat{\mathrm {PI}}(U;O)$. A plot of the mean and standard deviation traces corresponding to our 7 different users (similar to Figs. 3 and 4) is given in Fig. 12. And the evaluation of the partial PI estimates for each user (i.e., $\hat{\mathrm {PI}}(U=u;O)$) is given in Table 2.

Clearly, we see that the EEG signals are also (in fact even more) informative in this case. Interestingly, this observation is consistent with the related literature trying to exploit EEG signals for biometric applications [28, 29].

**Table 2:** Estimated PI values with maximum profiling set

User	$\hat{\mathrm {PI}}(U=u;O)$ with avg PCA	$\hat{\mathrm {PI}}(U=u;O)$ with raw PCA
1	0.7044	0.5257
2	0.7217	0.6378
3	0.2680	0.2138
4	0.3337	0.8044
5	$\varnothing$	$\varnothing$
6	0.2620	0.4254
7	0.4003	0.5650
8	1.4532	1.1351

## Consequences and conclusions

The results in this paper lead to two conclusions.

First, and from the security point of view, our experiments show that PIN extraction attacks using BCIs are feasible, yet require several observations to succeed with high probability. In this respect, the difference between the complexity of successful supervised/profiled attacks (around 10 correct PIN observations) and unsupervised/non-profiled attacks (more in the hundreds range) is noticeable. It suggests the aggregation of users into classes for which the models are sufficiently similar as an interesting scope for further research (which would require larger scale experiments, with more users). In this setting, a better investigation of the impact of enumeration would also be worthwhile. Indeed, the reduction of the average rank of the correct PIN is also significant in our analyses. Therefore, combining side-channel attacks against the human brain with some enumeration power can reduce the number of observations required to succeed. (Roughly, we can assume that the average key rank will be reduced exponentially in the number of observations, as usually observed in side-channel attacks [30].)

More generally, our results suggest that extracting concrete PIN codes from EEG signals, while theoretically feasible and potentially damaging from some users and PINs, is not yet a very critical threat for systematic PIN extraction. This may change in the future, if/when massive amounts of BCI signals start to be collected. Besides, other targets with smaller cardinality could already be more worrying (e.g., extracting the knowledge of one relative among a set of unknown people displayed on a screen), because of avoiding issues related to users loosing their focus due to too long experiments.

Second, and given the importance of profiling for efficient information extraction from EEG signals, our experiments also underline that privacy issues may be even more worrying than security ones in BCI-based applications. Indeed, when it comes to privacy, the adversary trying to identify a user is much less limited in his profiling abilities. In fact, any correlation between his target user and some feature found in a dataset is potentially exploitable. Furthermore, the amount and types of correlations that can be exhibited in this case are potentially unbounded, which makes the associated risks very hard to quantify. In this respect, the data minimization principle does not seem to be a sufficient answer: it may very well be that the EEG signals collected for one (e.g., gaming) activity can be used to reveal various other types of (e.g., medical, political) correlations. Anonymity is probably not the right answer either (since correlations with groups of users may be as discriminant as personal ones). And such issues are naturally amplified in case of malicious applications (e.g., it seems possible to design a BCI-based game where situations lead the users to incidentally reveal preferences). So overall, it appears as an important challenge to design tools that provide evidence of “fair treatment” when manipulating sensitive data such as EEG signals, which can be connected to emerging challenges related to computations on encrypted data [31] which can be connected to emerging challenges related to computations on encrypted data [31].

## Appendix 1: Gaussian template results

See Table 3.

**Table 3:** Gaussian counterpart to Table 1

User	$\hat{\mathrm {PI}}(P;O)$ with avg. PCA	$\hat{\mathrm {PI}}(P;O)$ with raw PCA
1	0.0795	0.0563
2	0.1454	0.1522
3	0.1542	0.1247
4	0.0824	0.0256
5	$\varnothing$	$\varnothing$
6	0.0586	0.0200
7	0.0841	0.0633
8	0.1752	0.0364

## Appendix 2: Additional figures

See Figs. 13, 14, 15, 16 and 17.

## Footnotes

1 The experiments described next were approved by the local Research Ethics Committee and performed in compliance with the Code of Ethics of the World Medical Association (Declaration of Helsinki). All participants gave written informed consent.

2 We further checked systematically that other electrodes did not provide significantly more discriminating information so that our conclusions would be affected.

3 Since we used the small sample size PCA variant in [19].

4 Note that for completeness, we also considered simple Gaussian templates. Comparing nonparametric and parametric approaches was useful in our experiments, in order to gain confidence that the kernel density estimation is not capturing dataset-specific features. Yet, since no significant variation was noticed, the following sections will focus on the results obtain with kernel density estimation.

5 More precisely, the PI is an average metric, so what is needed is that each line of the PI matrix defined in [17] (corresponding to 6 different events in our study) are positive, which we confirmed with the success rate analysis.

6 We note that confidence intervals estimated based on a Gaussian assumption did not lead to different conclusions in our case study.

7 As mentioned in Sect. 2, this is due to the presence of another familiar event for this user, which he mentioned to us after the experiments were performed. Further analysis of this critical case was not possible since the experiment approved by our ethical board was conditioned on the fact that no user PIN was stored.

8 We may expect more singularities (such as the one of User 5) to appear and launch false alarms in case studies with more PIN values. Yet, this would not contradict the trend of a significantly reduced average rank for the correct PIN value.

9 Despite a positive PI, the key rank for User 7 also stabilizes to 2. Yet, in this case we observed that it is due one single misleading observation that is not rejected by our outlier management).

10 Excluding User 5 because of its previously computed negative PI.
